# Imaging Findings of Adenocarcinoma Arising from Lingual Foregut Duplication Cyst

**DOI:** 10.5334/jbsr.3156

**Published:** 2023-08-25

**Authors:** Jung Youn Kim, Na Yeon Yoon, Sung-Eun Choi

**Affiliations:** 1 CHA Bundang Medical Center, KR; 2 Department of Radiology, CHA Bundang Medical Center, KR; 3 Department of Pathology, CHA Bundang Medical Center, KR

**Keywords:** head and neck, head and neck imaging, tongue, lingual, foregut duplication cyst, adenocarcinoma

## Abstract

Lingual foregut duplication cysts are rare developmental anomalies with varied symptoms, and adenocarcinomas may develop from them. Radiological evaluations, including computed tomography and magnetic resonance imaging, can help diagnose these lesions and determine their extent. Abnormal enhancement should raise the suspicion of malignancy of a lingual foregut duplication cyst. Early detection and complete surgical resection of lingual foregut duplication cysts are necessary to prevent malignant transformations. Complete tumor resection with adjuvant radiotherapy or radiochemotherapy is recommended. The teaching point is that mucinous adenocarcinoma can develop from a lingual foregut duplication cyst, and radiological studies may aid in the diagnosis.

**Teaching Point:**

Adenocarcinomas can develop from lingual foregut duplication cysts, which are rare developmental anomalies, and radiologic studies may aid in the diagnosis.

## Introduction

Congenital cystic lesions with foregut-derived epithelial linings, known as foregut duplication cysts, can manifest anywhere from the oral cavity to the anus [1]. Lingual foregut duplication cysts are rare and account for only 0.3% of all diagnosed foregut cysts [2]. Adenocarcinomas rarely develop from lingual foregut duplication cysts, and reports on their radiologic findings are limited.

## Case History

A 63-year-old male presented to the outpatient clinic of the otorhinolaryngology department with complaints of the sensation of mouth fullness and dysphagia that had lasted for three months. On physical examination, the tongue showed bulging contour with a normal overlying mucosa.

**Figure 1:**
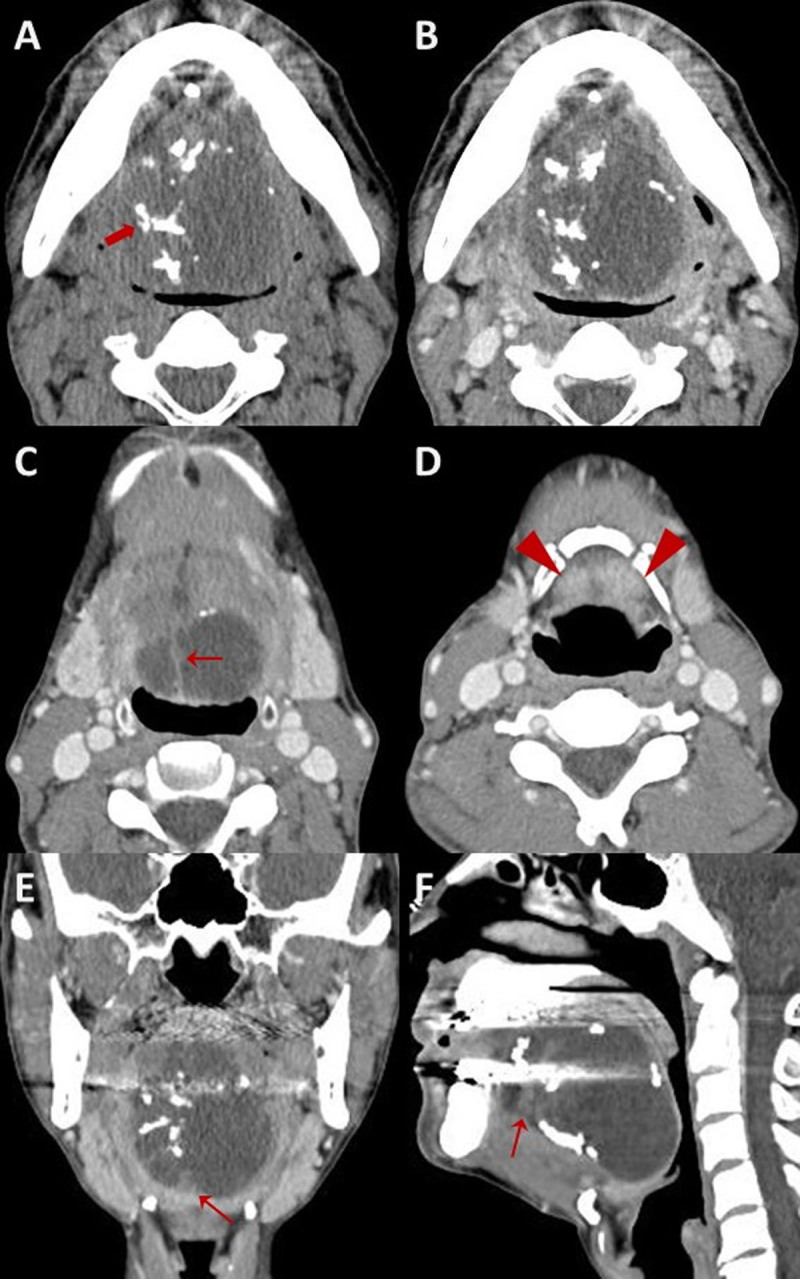
Neck CT showing a low-attenuation mass in the tongue with internal calcifications **(thick arrow in A)**. The contrast-enhanced images demonstrate septa-like structures within the cystic mass **(thin arrows in C, E, and F)**. The bilateral sublingual glands are displaced inferiorly by the mass **(arrowhead in D)**.

**Figure 2:**
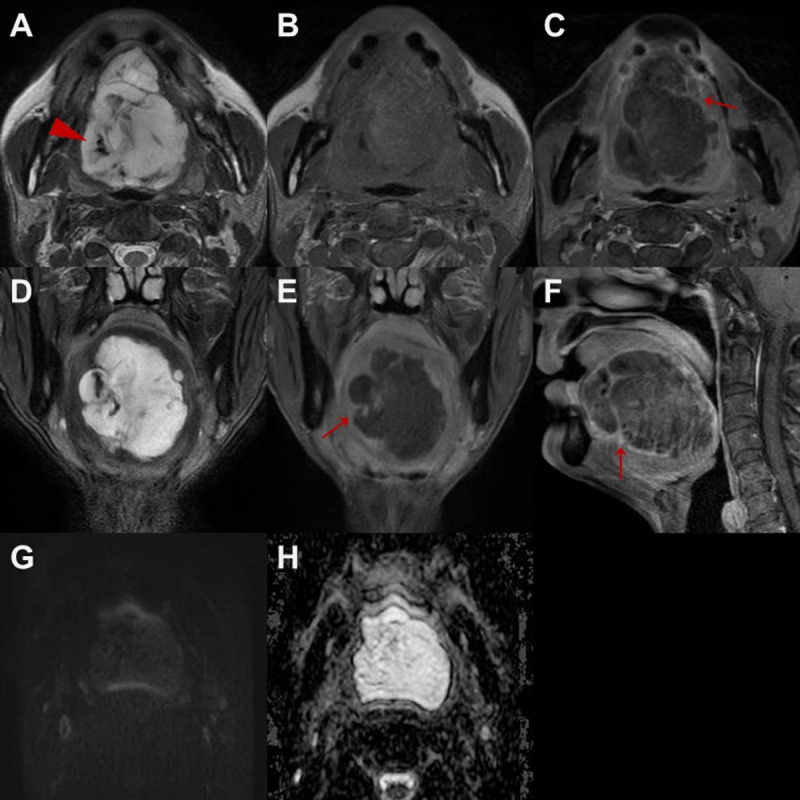
Neck MRI showing a cystic mass that is hyperintense on T2WI **(A)** and isointense on T1WI **(B)** with internal calcifications observed as punctate hypointensities on T2WI (arrowhead in A). The contrast-enhanced T1WIs reveal thin enhancements at the internal septations and peripheral wall of the mass **(arrows in C, E, and F)**. The fat-suppressed T2WI show no evidence of fat components in the mass (D). The diffusion-weighted and apparent diffusion coefficient images show no significant diffusion restriction **(G and H)**.

Enhanced neck computed tomography (CT) and magnetic resonance imaging (MRI) revealed a well-circumscribed lobulated cystic mass in the tongue, measuring approximately 6.7 × 5.7 × 5.1 cm. CT images showed a low-attenuation mass with multifocal internal calcifications, and septa-like structures were observed within the mass (Figure 1). On neck MRI, the mass was hyperintense on T2-weighted imaging (T2WI) and isointense on T1-weighted imaging (T1WI) with thin enhancing internal multiseptations (Figure 2). There was no evidence of pathological lymph node in the neck.

**Figure 3:**
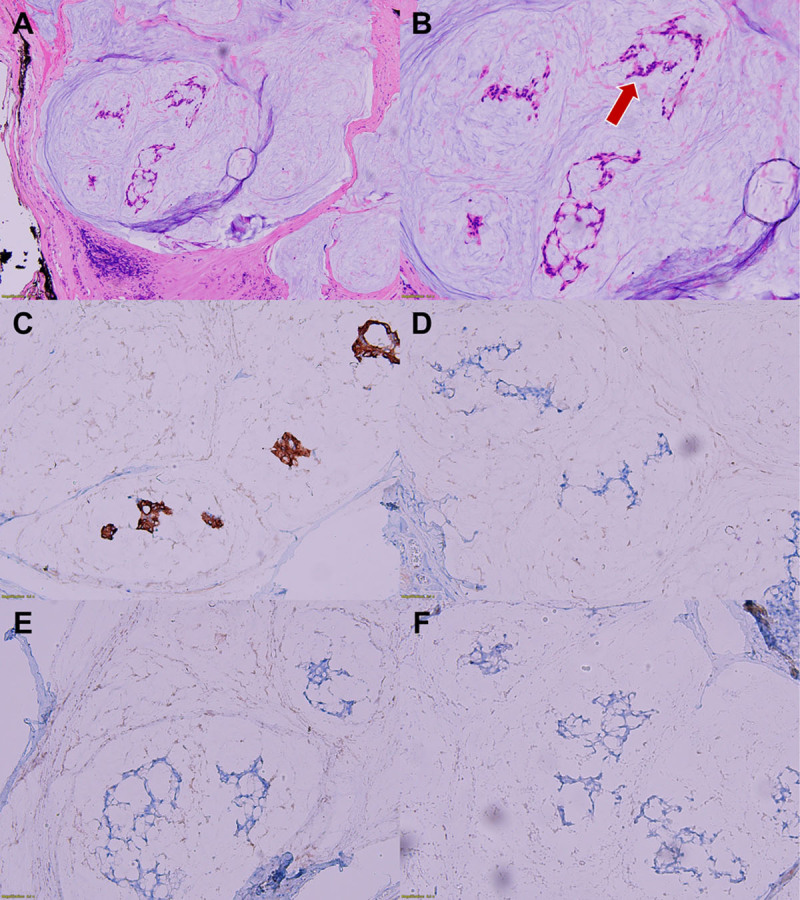
Histopathological examination reveals abundant mucin pools lined with goblet cells, suggestive of intestinal-type epithelium. Floating epithelial cells with areas of nuclear dysplasia and transition to adenocarcinomas are observed within the mucin on hematoxylin-eosin staining **(arrow in B)**. The tumor cells show strong positive staining for CK20 **(C)** and negative staining for CK7 **(D)**, AR **(E)**, and P63 **(F)**: magnification ×100 (A) and ×200 **(B–F)**.

The initial radiologic diagnosis of this mass was a lingual foregut duplication cyst, with differential diagnoses of lymphatic malformation and dermoid cyst. Given the impression of a benign mass, the surgeon proceeded with trans-oral resection surgery. After histopathological analysis of the excised mass, the final diagnosis was mucinous adenocarcinoma (Figure 3), and tumor involvement at the resection margin was positive.

**Figure 4:**
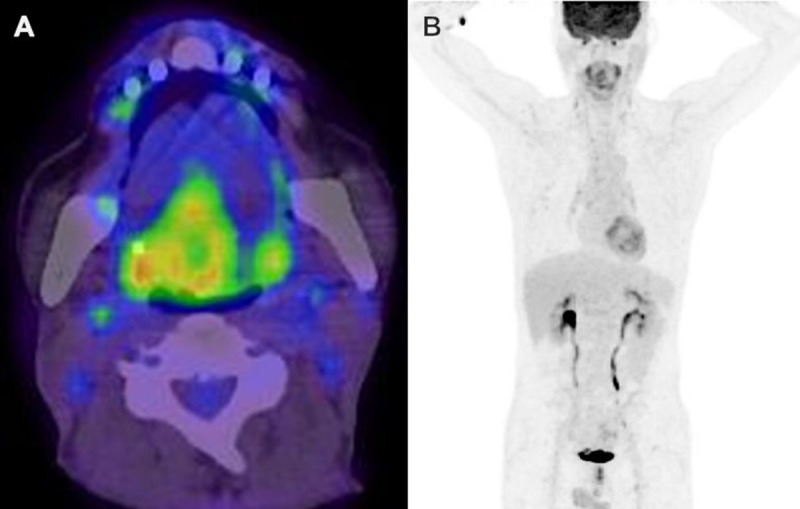
The positron emission tomography/CT images show no evidence of increased uptake in any location, except at the resection margin of the tongue.

The patient underwent positron emission tomography/CT scan to exclude the possibility of a metastatic lesion from a primary intestinal malignancy, and there was no evidence of a metabolically active lesion in any location other than the resection margin of the tongue (Figure 4). Upper endoscopy and colonoscopy were performed; however, no abnormal findings were observed.

After a multidisciplinary discussion, total glossectomy was planned as a treatment plan, and adjuvant radiochemotherapy was considered a subsequent treatment regimen.

## Comments

Lingual foregut duplication cysts are uncommon anomalies that occur as a result of remnant heterotopic rests of the foregut-derived epithelium after the first trimester of embryological development [1]. Although exceedingly rare, adenocarcinomas can develop from foregut duplication cysts and have mostly been reported in alimentary organs [3,4]. There have been reported cases of primary intestinal-type adenocarcinoma developing on the tongue; however, most are believed to originate from minor salivary gland tissues [5,6]. To the best of our knowledge, this is the second reported case of adenocarcinoma arising from a lingual foregut duplication cyst [7]. Therefore, this report may provide evidence that emphasizes the need for early detection and resection of lingual foregut duplication cysts to prevent malignant transformation.

Radiologic evaluations can help diagnose these lesions and determine their extent. In our case, enhancements of the peripheral wall and internal septations of the lingual foregut duplication cyst were observed on radiologic images, and these imaging findings may reflect the malignant transformation of foregut duplication cyst.

Although no standardized treatment protocol has been established for adenocarcinomas arising from foregut duplication cysts because of their rarity, complete resection with adjuvant radiotherapy or radiochemotherapy is recommended [5].
